# MicroRNA deep sequencing in two adult stem cell populations identifies miR-501 as a novel regulator of myosin heavy chain during muscle regeneration

**DOI:** 10.1242/dev.136051

**Published:** 2016-11-15

**Authors:** Amir Mizbani, Edlira Luca, Elisabeth J. Rushing, Jan Krützfeldt

**Affiliations:** 1 Division of Endocrinology, Diabetes, and Clinical Nutrition, University Hospital Zurich, Zurich 8091, Switzerland; 2 Competence Center Personalized Medicine UZH/ETH, ETH Zurich and University of Zurich, Zurich, Switzerland; 3 Institute of Neuropathology, University Zurich and University Hospital Zurich, Zurich 8091, Switzerland; 4 Zurich Center for Integrative Human Physiology, University of Zurich, Zurich, Switzerland

**Keywords:** MicroRNA, Skeletal muscle, Regeneration, Myosin, Serum

## Abstract

MicroRNAs (miRNAs) are important regulators of skeletal muscle regeneration, but the underlying mechanisms are still incompletely understood. Here, comparative miRNA sequencing analysis of myogenic progenitor cells (MPs) and non-myogenic fibroblast-adipocyte progenitors (FAPs) during cardiotoxin (CTX)-induced muscle injury uncovered miR-501 as a novel muscle-specific miRNA. miR-501 is an intronic miRNA and its expression levels in MPs correlated with its host gene, chloride channel, voltage-sensitive 5 (*Clcn5*). Pharmacological inhibition of miR-501 dramatically blunted the induction of embryonic myosin heavy chain (MYH3) and, to a lesser extent, adult myosin isoforms during muscle regeneration, and promoted small-diameter neofibers. An unbiased target identification approach in primary myoblasts validated gigaxonin as a target of miR-501 that mimicked the effect of miR-501 inhibition on MYH3 expression. In the *mdx* mouse model, which models a pathological disease state, not only was miR-501 induced in regenerating skeletal muscle, but also its serum levels were increased, which correlated with the disease state of the animals. Our results suggest that miR-501 plays a key role in adult muscle regeneration and might serve as a novel serum biomarker for the activation of adult muscle stem cells.

## Abstract (precis)

**Summary:** MicroRNA 501 is a novel muscle-specific microRNA that is induced during muscle regeneration and regulates the transition of myosin heavy chains during early myogenesis.

## INTRODUCTION

Skeletal muscle function is crucial for the maintenance of health. It has been estimated that about 80% of cancer patients experience muscle wasting, and that the loss of muscle mass contributes significantly to decreased quality of life or even death (Cohen et al., 2015). Loss of muscle mass can be caused by factors intrinsic to adult myofibers such as degradation of myofibrillar proteins by the ubiquitin-proteasome pathway (Cohen et al., 2009, 2012). However, muscle mass is also determined by the capacity of skeletal muscle to regenerate. Regeneration of skeletal muscle depends on the activation of myogenic progenitors (MPs), and their proliferation and differentiation into committed myoblasts, which finally fuse to build or repair myofibers. Activation of inflammatory pathways in MPs has been shown to contribute to muscle wasting in cachexia (He et al., 2013). Transplantation of MPs in aged mice was able to prevent muscle loss (Hall et al., 2010) and improving the regenerative capacity of MPs during aging improved muscle strength (Cosgrove et al., 2014). Skeletal muscle regeneration resembles the myogenesis process during development in many aspects, such as the expression of developmental myosins. The embryonic isoform of myosin, MYH3, is expressed during the early phase of regeneration and is slowly replaced in the course of the following 2-3 weeks by the adult myosin heavy chain (MHC) isoforms (Schiaffino et al., 2015). This renders developmental myosin a marker for skeletal muscle regeneration in both animal models and human myopathies including rhabdomyosarcoma (Schiaffino et al., 1986). The switch from embryonic myosin to type II fast adult MHCs is nerve independent, whereas the switch to type I MHCs is predominantly driven by nerve activity (d'Albis et al., 1988). The regulation of the initial expression of developmental and adult myosins during muscle regeneration is only poorly understood.

MicroRNAs (miRNAs) are a class of short non-coding RNAs of 20-24 nucleotides in length. Several miRNAs specific to striated muscle, also referred to as myomiRs, have been described to date, such as miR-1, -133a/b, -206, -208a/b and -499 (Chen et al., 2009), and have been shown to regulate myogenesis. Most myomiRs are expressed in both cardiac and skeletal muscle, although miR-208a and miR-206/miR-133b are specific to cardiac and skeletal muscle, respectively (Chen et al., 2009). miR-1 and miR-206 partake in promoting differentiation over proliferation of stem cells. miR-1 targets histone deacetylase 4, which is a repressor of muscle differentiation (Chen et al., 2006), whereas miR-1 and miR-206 repress paired box 7, an essential factor for the muscle differentiation process (Chen et al., 2010). miR-206 is also involved in terminal differentiation of myoblasts by targeting connexin-43 (also known as gap junction protein, alpha 1) (Anderson et al., 2006) and in the inhibition of cell proliferation by targeting the p180 subunit of DNA polymerase-α (Kim et al., 2006). Despite the absence of an overt skeletal muscle phenotype, *miR-206* knockout mice show a remarkably delayed regenerative response to cardiotoxin (CTX)-induced muscle injury (Liu et al., 2012). When crossed to the muscle dystrophy mouse model *mdx*, it was observed that lack of miR-206 aggravates the dystrophic phenotype (Liu et al., 2012). In contrast to miR-1 and miR-206, miR-133 inhibits the differentiation of myoblasts and promotes their proliferation by repressing serum response factor (Chen et al., 2006). Interestingly, the miR-208 family, including miR-208a/b and miR-499, are all intronic miRNAs located and co-transcribed with various myosin genes. miR-208a is encoded within the *Myh6* gene (α-myosin heavy chain) and is expressed specifically in cardiac muscle (van Rooij et al., 2007), whereas miR-208b and miR-499 are processed from introns in *Myh7* (β-myosin heavy chain) and *Myh7b* (myosin heavy chain 7b) genes, respectively (Chen et al., 2009). Functioning in a redundant manner owing to identical seed sequences, the last two miRNAs control the fiber type switch in skeletal muscle by targeting *Sox6*, *Sp3*, *Purb* and *HP-1b* (haptoglobin) (van Rooij et al., 2009). Mice lacking both miR-208b and miR-499 have substantially fewer type I fibers in soleus muscle, whereas overexpression of miR-499 in soleus leads to the switch of fast type II myofibers to slow type I myofibers (van Rooij et al., 2009). Whether a muscle-specific miRNA regulates myosin formation in regenerating skeletal muscle is still unknown.

Here, we report a novel muscle-specific miRNA, miR-501, located in an intron of isoform-2 of the *Clcn5* gene and expressed specifically in activated MPs and newly formed myofibers. miR-501 induction in activated muscle stem cells parallels the progression of muscle regeneration processes and regulates the expression of MHC in newly formed fibers. Furthermore, surges in miR-501 levels in serum of *mdx* mice correlate with the regenerative phases in muscle. Overall, we propose miR-501 as a new myomiR and biomarker of MP activation that regulates the transition of embryonic myosin heavy chain to adult isoforms during skeletal muscle regeneration.

## RESULTS

### microRNA deep sequencing in two adult stem cell populations

**Fig. 1.**
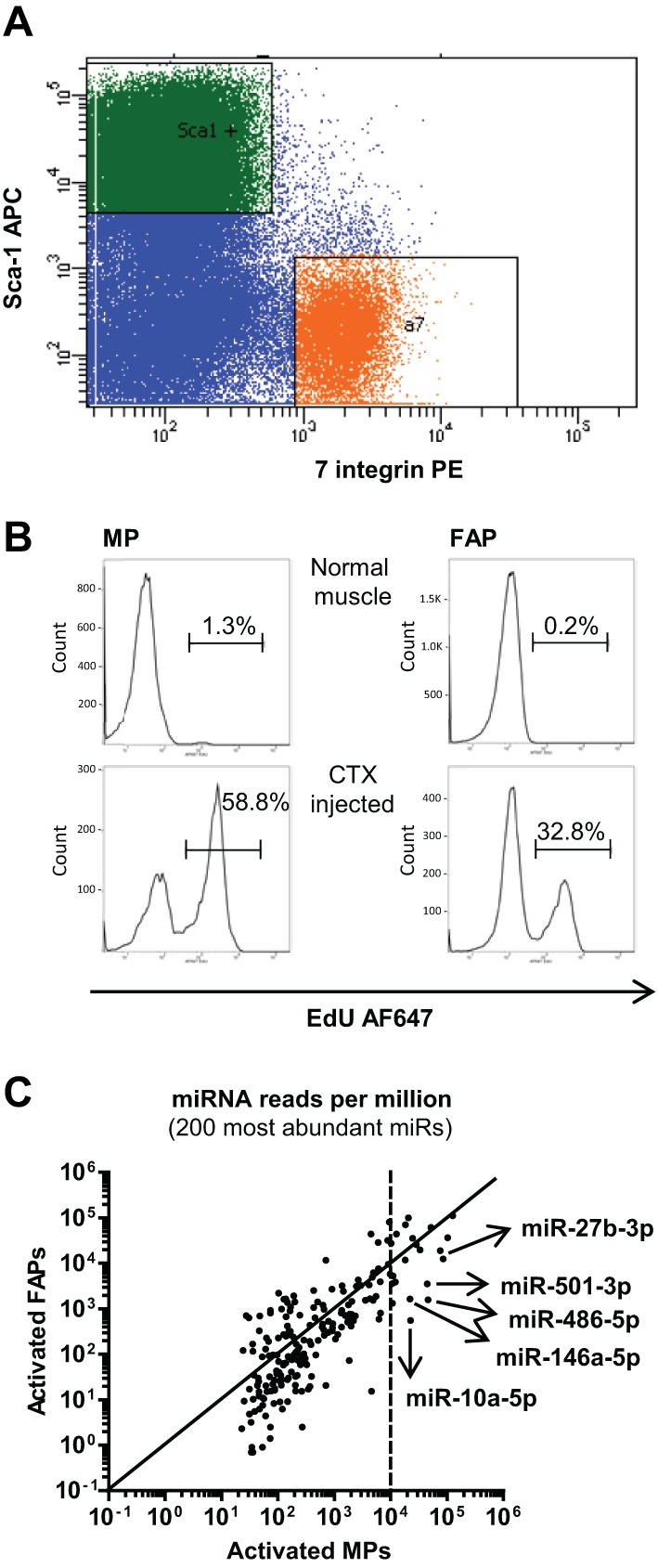
**miR-501 is highly enriched in activated myogenic progenitors.** TA muscles were injected with CTX and analyzed 3 days later. (A) MPs and FAPs were identified based on their distinct expression pattern of SCA1 and alpha 7-integrin. Orange: PE^+^ gate; green: APC^+^ gate. (B) Mice were injected intraperitoneally with 10 µg EdU per gram body weight 12 h before harvesting. Proliferation was measured by EdU incorporation assay using FACS. Shown are cell counts based on Alexa Fluor 647 (AF647) staining. (C) Results of small RNA deep sequencing, comparing activated MPs and FAPs, *n*=2, each sample representing a pool of cells obtained from seven to nine mice for MPs and two mice for FAPs. The five miRNAs that were identified as being highly expressed and at least fivefold enriched in MPs versus FAPs are marked.

**Fig. 2.**
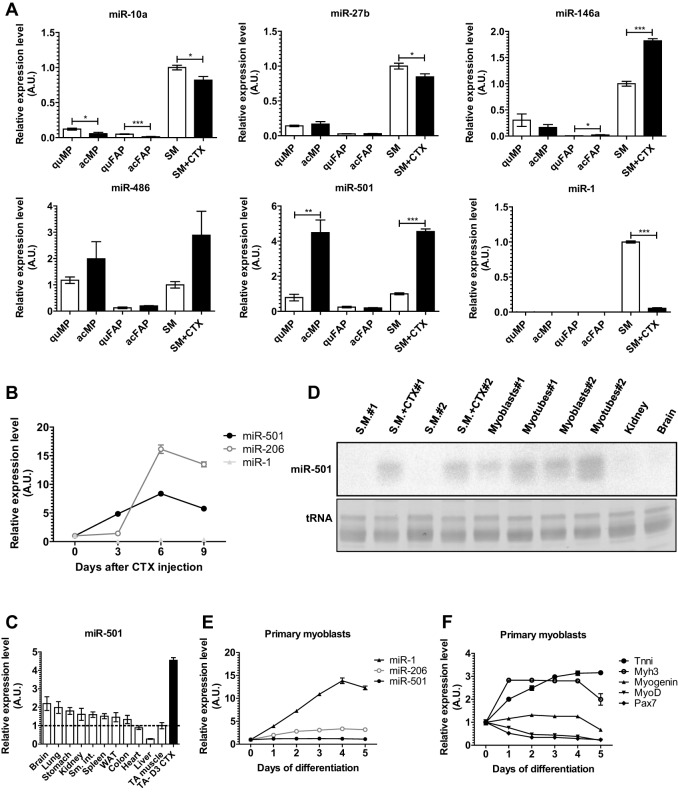
**miR-501 expression is specific to myogenic progenitors and induced during skeletal muscle regeneration.** (A) Expression of the selected miRNAs in MPs and FAPs sorted from normal (SM) or CTX-injected (SM+CTX) TA skeletal muscle, measured by qRT-PCR. miR-1 was measured as an adult-muscle-specific miRNA. *n*=4-6. acFAP, activated FAPs; acMP, activated MPs; quFAP, quiescent FAPs; quMP, quiescent MPs. (B) Expression of miR-501, miR-206 and miR-1 up to 9 days after CTX injection, measured as in A. *n*=3 for each time point. (C) Expression of miR-501 in different mouse tissues measured by qRT-PCR. *n*=5 for TA-CTX, *n*=3 for the other tissues. D3 CTX, day 3 after CTX treatment; Sm. Int., small intestine; WAT, white adipose tissue. (D) Confirmation of tissue-specific miR-501 expression in CTX-injected TA, primary myoblasts, and myotubes by northern blotting. Ethidium bromide staining of tRNA is shown as loading control. (E,F) The indicated muscle-specific miRNAs (E) or muscle differentiation markers (F) were measured during differentiation of mouse primary myoblasts after serum withdrawal for the indicated time points. *n*=3. Data were normalized to sno234 in A,B,C and E, and to 18S RNA in F and presented as mean±s.e.m. relative to normal TA muscle or undifferentiated myoblasts. *P<0.05, **P<0.01, ***P<0.001, Student's *t*-test. CTX, cardiotoxin; SM, skeletal muscle.

To identify a novel muscle-specific miRNA, we analyzed miRNA expression in activated MPs compared with a non-myogenic cell population, fibrocyte-adipocyte progenitors (FAPs). Activation of MPs was achieved by a single intramuscular CTX injection. Previous studies have indicated that the proliferation response is maximal 3 days after CTX injection (Joe et al., 2010). Indeed, we observed that a single pulse of 5-ethynyl-2′-deoxyuridine (EdU) labeled ∼60% of MPs and ∼30% of FAP cells (Fig. 1A,B). As expected, MPs or FAPs from uninjected muscle showed only minor proliferation activity (Fig. 1B). To identify the miRNAs specifically expressed in MPs, we collected MPs and FAPs using fluorescence-activated cell sorting (FACS) for alpha7-integrin^+^SCA1^−^lin^−^ cells or alpha7-integrin^−^SCA1^+^lin^−^ cells, respectively, 3 days after CTX application and isolated RNA for Illumina deep sequencing of miRNAs. Overall, we detected 478 and 535 miRNA sequences in two independent biological replicates of activated MPs. Five miRNAs were highly expressed representing at least 1% of all retrieved miRNA sequences and were at least fivefold enriched in MPs versus FAPs (Fig. 1C; Table 1). Importantly, all five miRNAs were conserved between mice and humans (mirbase.org). Next, we compared the expression of these miRNAs as well as miR-1 between quiescent and activated MPs and FAPs and adult skeletal muscle using qRT-PCR (Fig. 2A). Consistent with our sequencing data, miR-1 was expressed at a low level in MPs and FAPs compared with adult skeletal muscle, and the five selected miRNAs had higher expression in activated MPs compared with activated FAPs. However, only miR-501 had substantially higher expression in activated versus quiescent MPs as well as in activated MPs compared with normal adult skeletal muscle. These results indicated that miR-501 in skeletal muscle is induced specifically in activated MPs during muscle regeneration.

**Table 1.**
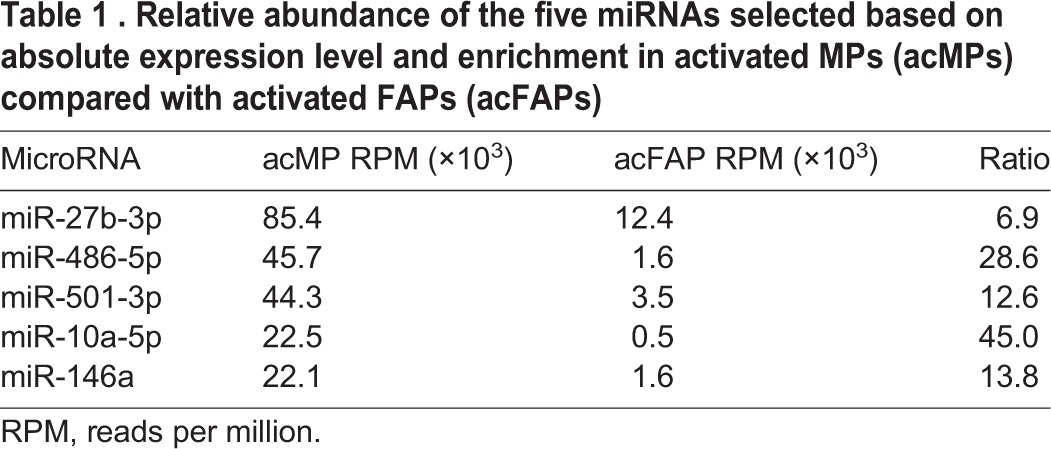
**Relative abundance of the five miRNAs selected based on absolute expression level and enrichment in activated MPs (acMPs) compared with activated FAPs (acFAPs)**

### Expression of miR-501 and its host gene isoform-2 of Clcn5 in regenerating skeletal muscle

To improve our understanding of the regulation of miR-501 during muscle regeneration, we analyzed the kinetics of miR-501 at different time points after CTX-induced muscle injury. We also analyzed expression of miR-1, which is known to be a marker of adult non-regenerative muscle, and miR-206, which is induced during muscle differentiation (Liu et al., 2012). As expected, miR-1 was expressed at a low level during regeneration whereas miR-206 expression was strongly induced 6 days after CTX injection (Fig. 2B). Expression levels of miR-206, however, remained unchanged during the first 3 days of muscle regeneration when MP proliferation is maximally stimulated. By contrast, miR-501 expression was immediately upregulated after muscle injury, providing a more accurate representation of the progression of muscle regeneration (Fig. 2B). We then determined the expression of miR-501 by qRT-PCR in various mouse tissues and identified regenerating skeletal muscle as the tissue with the highest expression of this miRNA (Fig. 2C). Northern blotting confirmed expression of miR-501 in regenerating muscle as well as in primary myoblasts and myotubes (Fig. 2D). Importantly, miR-501 was not detected in non-regenerating muscle, adult kidney or brain (Fig. 2D). No major regulation was observed for miR-501 during differentiation of myoblasts in contrast to miR-1 (Fig. 2D-F). Together, these data identify miR-501 as a myomiR specific to adult muscle stem cells and one which is induced specifically during the activation of these cells in the process of regeneration.

**Fig. 3.**
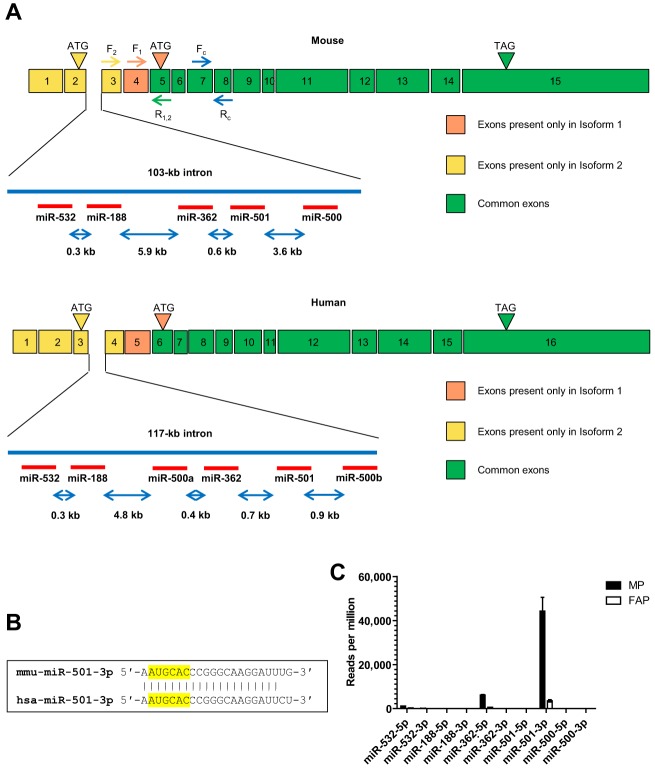
**miR-501 is a conserved intronic miRNA residing in the *Clcn5* gene, isoform-2.** (A) Schematic of mouse (top) and human (bottom) *miR-501* locus and its host gene, *Clcn5*. The intron located between two isoform 2-specific exons harbors a cluster of conserved miRNAs. Binding sites for isoform-specific (F_1_, F_2_, R_1,2_) and common primers (F_C_, R_C_) used for mouse transcripts are indicated. (B) Alignment of mouse and human miR-501-3p sequences. The seed region is highlighted. (C) Relative expression of the miRNAs clustered in intron 2 of mouse *Clcn5* in MPs and FAPs sorted from CTX-injected TA muscle, as shown in Fig. 1. Data are the mean±s.e.m.

To address the mechanisms that drive the expression of miR-501 in muscle precursor cells, we looked into the genomic residence of this miRNA. Intriguingly, miR-501 is located in an intron of the chloride channel 5 gene (*Clcn5*) in a cluster with other miRNAs (Fig. 3A). An alternative splicing upstream of exon 5 leads to the generation of two splice variants of *Clcn5* in both mouse and humans (NCBI RefSeq). In mouse, splice variant 1 (NM_016691) encodes a 746-amino acid protein with a start site at exon 4, whereas splice variant 2 (NM_001243762) includes exons 1, 2 and 3, hence encoding a protein with 70 additional amino acids at its N terminus. Interestingly, the intron harboring the miRNA cluster is located between exons 2 and 3 in mouse and 3 and 4 in human, which are present only in isoform-2 of *Clcn5*. Alignment of mouse and human miR-501-3p sequences indicate a high degree of conservation (Fig. 3B). We returned to our RNA deep sequencing data to analyze the expression of the other intronic miRNAs in MPs. However, we observed that only miR-501 and its closest neighbor miRNA, miR-362, were detectable (Fig. 3C). Possibly, post-transcriptional mechanisms might dictate the preferential expression of miR-501 compared with the other intronic miRNAs.

**Fig. 4.**
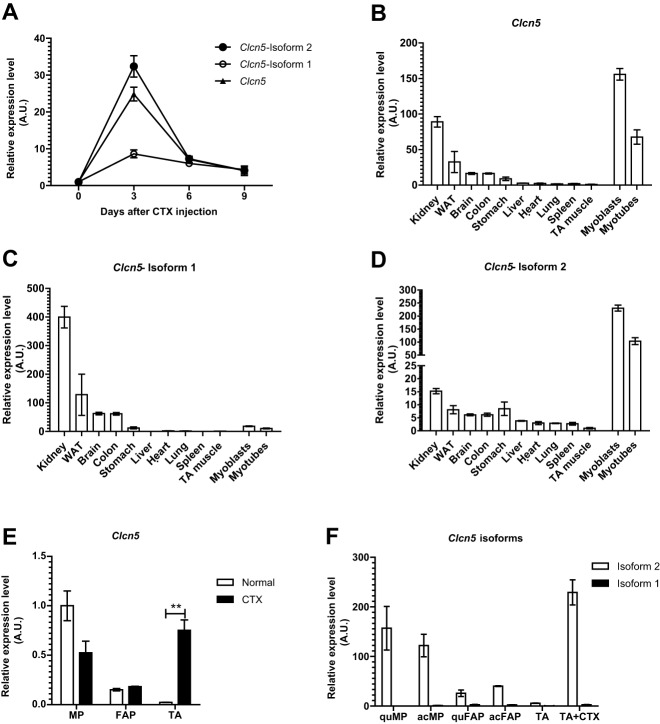
**Upregulation of *Clcn5* expression during skeletal muscle regeneration and its enrichment in myogenic progentor cells is specific to isoform 2.** (A) Expression of *Clcn5* isoforms in regenerating TA muscle as measured by qRT-PCR using isoform-specific primers, as depicted in Fig. 3A. F_1_ and F_2_ forward primers were used to specifically measure isoforms 1 and 2, respectively, and R_1,2_ was used as reverse primer for both. *Clcn5* (triangles) corresponds to the whole *Clcn5* transcripts, measured by using F_C_ and R_C_ primers. *n*=3. (B-D) Expression of *Clcn5* and its isoforms in different mouse tissues was measured by qRT-PCR. *n*=3. WAT, white adipose tissue. (E,F) Progenitor cells were sorted by FACS from normal or CTX-injected TA muscle (day 3), and the expression of *Clcn5* (E) and its isoforms (F) was measured by qRT-PCR using isoform-specific primers. Each sample was composed of cells sorted from three or four mice. qPCR data in all panels was normalized to 18S rRNA. Data are presented as mean±s.e.m. **P<0.01, Student's *t*-test. acFAP, activated FAPs; acMP, activated MPs; quFAP, quiescent FAPs; quMP, quiescent MPs.

Isoform-1 of the *Clcn5* gene encodes a Cl^−^/H^+^-exchanger highly expressed in kidney, where it plays a crucial role in the process of endocytosis in the proximal tubule (Piwon et al., 2000; Christensen et al., 2003). We analyzed the expression of both *Clcn5* isoforms during skeletal muscle regeneration. Intriguingly, we observed that isoform-2, but not isoform-1, is highly induced (Fig. 4A). Whereas we could confirm that isoform-1 expression is highest in kidney (Fig. 4B,C), we observed that isoform-2 expression is specific to muscle precursor cells (Fig. 4D). Furthermore, in progenitor cells isolated from adult skeletal muscle, *Clcn5* isoform-2 is the dominant isoform with comparable expression levels in activated and quiescent MPs (Fig. 4E,F). This expression pattern indicates a common promoter element shared by *Clcn5* isoform-2 and miR-501. However, the strong induction of miR-501 upon activation of MPs is not observed for *Clcn5* isoform-2 and indicates additional regulatory steps at the post-transcriptional level of the miR-501 precursor.

### Silencing of miR-501 *in vivo* prevents myosin heavy chain expression during muscle regeneration

**Fig. 5.**
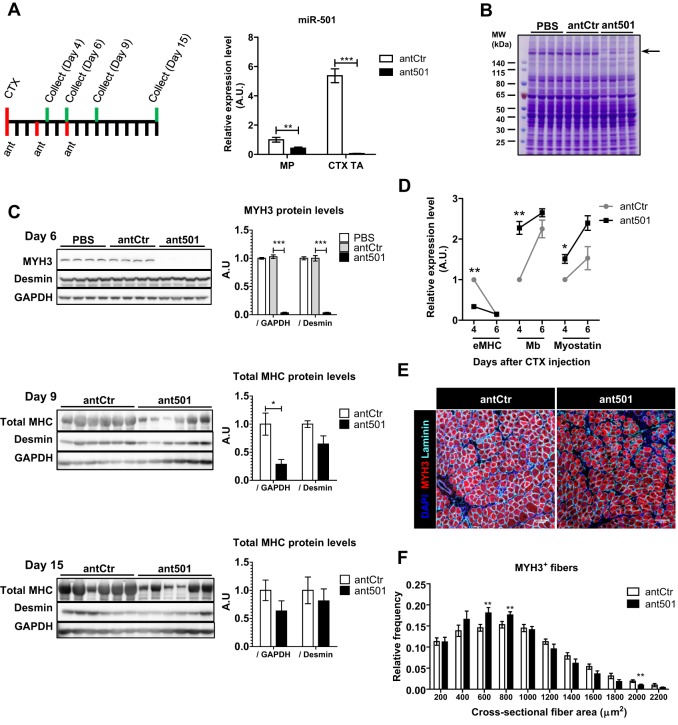
**Inhibition of miR-501 in regenerating skeletal muscle inhibits appearance of myosin heavy chain and formation of large myofibers.** (A) Mouse TA muscles were injected with CTX and control antagomir (antCtr) or antagomir-501 (ant501). Antagomir injection was repeated after 3 and 6 days, and muscles were harvested on day 4, 6, 9 or 15. qRT-PCR analysis of miR-501 expression is shown for FACS-isolated MPs or regenerating muscle tissue (CTX TA) at day 4 (*n*=4-6, normalized to sno234). (B) Coomassie Blue staining is shown after resolving muscle lysates harvested on day 6 from PBS, antCtr and ant501 treated animals. (C) Western blot analysis for MYH3 and desmin proteins from one TA muscle on day 6 of regeneration (*n*=4 mice per group) or for total MYH on day 9 and 15 after CTX injection from both TA muscles (*n*=3 mice). Bar graphs show densitometry of western blots normalized to GAPDH or desmin, as indicated. (D) Expression of *Myh3* (eMHC) and adult muscle markers myoglobin (Mb) and myostatin transcripts in TA muscle on day 4 and 6 after CTX injection as measured by qRT-PCR. Data are normalized to 18S rRNA and shown relative to day 4 in antCtr group. *n*=2-3 mice, 4-6 TA muscles per group and time point. (E,F) Frozen muscle sections harvested on day 6 after CTX were probed with MYH3- and laminin-antibodies, and DAPI. Representative pictures are shown in E. Fiber diameter was analyzed based on laminin immunofluorescence and shown relative to the total number of fibers in F. *n*=7-8 mice per group. All data are presented as mean±s.e.m. *P<0.05, **P<0.01, ***P<0.001.

Our next aim was to determine the function of miR-501 during the process of muscle regeneration. For the inhibition of miR-501 *in vivo*, we injected antagomirs against miR-501, scrambled control antagomirs or PBS intramuscularly. Injection of antagomir-501 markedly reduced miR-501 levels in both MPs and regenerating muscle (Fig. 5A). Using EdU incorporation assays and flow cytometry analysis, we did not observe any change in proliferation rate of MPs 4 days after the induction of muscle injury (data not shown). Interestingly, muscle protein lysates separated by SDS-PAGE and stained with Coomassie Blue revealed a remarkable and specific decrease in dye intensity at the high molecular weight range (Fig. 5B), suggesting alteration of MHC levels. Indeed, western blot analysis for MYH3, the first type of MHC expressed in newly built fibers, revealed a dramatic decrease of this protein when miR-501 was inhibited (Fig. 5C). As muscle regeneration proceeds, MYH3 is replaced by other adult MHC isoforms. Western blot analysis of regenerating muscles at later time points also showed lower abundance of adult MHCs as measured with an antibody detecting all MHC isoforms after miR-501 was inhibited (Fig. 5C). Fifteen days after muscle regeneration was induced this difference was no longer present (Fig. 5C), and there was also no difference in muscle weight (data not shown). At the gene expression level, inhibiting miR-501 significantly blunts the induction of the *Myh3* transcript on day 4 of regeneration, but on day 6, *Myh3* expression in both control and antagomir-treated muscles returns to baseline (Fig. 5D). This indicates that the strong inhibition of MHY3 protein at day 6 occurs independently of transcription. In addition, we observed an upregulation of the adult muscle markers myoglobin and myostatin, suggesting that downregulation of MHC might not be a consequence of delayed differentiation. Immunofluorescence analysis of fiber size in the regenerating muscles demonstrated a significant shift towards smaller fibers after miR-501 inhibition (Fig. 5E,F). We conclude that miR-501 is crucial for MHC expression in newly formed myofibers mainly at the post-transcriptional level, hence its absence leads to a decrease in fiber size.

### Identification of miR-501 target genes

**Fig. 6.**
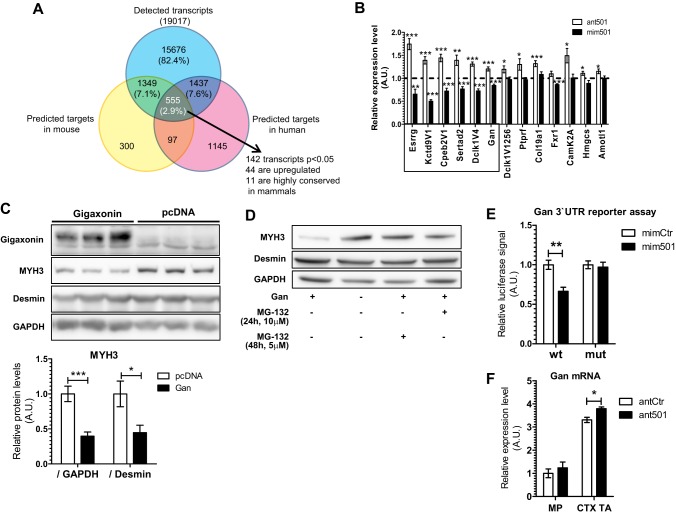
**Identification** **and validation of gigaxonin as a miR-501 target decreasing MYH3 levels in primary muscle cells.** Primary myoblasts were transfected with control antagomir or antagomir-501 and harvested after 48 h. RNA was extracted and used for cDNA synthesis and RNA-seq after DNase-treatment (*n*=3). (A) Venn diagram showing the overlap between predicted target genes for miR-501 in mouse and human, based on TargetScan v6.2. The 11 transcripts that were significantly upregulated, predicted as miR-501 targets in mouse and human, and conserved among mammals were considered for further analysis. (B) qRT-PCR confirmation of six out of the 11 selected genes as potential miR-501 targets based on inhibition or overexpression of miR-501 in primary myoblasts, respectively. Cells were harvested 48 h after transfection with the antagomirs or miRNA mimics. Values are shown relative to transfections with control mimic or antagomir as indicated by the dashed line. *n*=11-12. (C) Primary myoblasts were transfected with pcDNA3.1 vector encoding N-terminally FLAG-tagged gigaxonin or empty vector, and differentiation was induced by serum withdrawal for 2 days. Densitometry shows MYH3 protein normalized to GAPDH or desmin. *n*=6. (D) Effect of the proteasome inhibitor MG-132 on MYH3 levels after gigaxonin overexpression. MG-132 was added to the media at the indicated time points and concentrations before harvesting. (E) The human 3′ UTR of GAN was cloned into the pmirGLO vector with (mut) or without (wt) a mutation of three nucleotides in the miR-501-binding site. Constructs were transfected into HEK293 cells and luciferase activity was measured after 48 h. Firefly luciferase activity was normalized to *Renilla* luciferase activity. *n*=5. (F) qRT-PCR analysis of *Gan* expression in FACS-sorted MPs or regenerating muscle (CTX TA) 4 days after CTX injection. RNA derived from the same experiment shown in Fig. 5A. Data are presented as mean±s.e.m. All qRT-PCR data are normalized to 18S rRNA. *P<0.05, **P<0.1, ***P<0.001, Student's *t*-test.

In order to identify the relevant targets of miR-501, we performed quantitative analysis of the transcriptome in myoblasts with and without inhibition of miR-501. mRNA sequencing was performed on RNA isolated from primary myoblasts transfected with either control antagomir or antagomir-501. We aligned the list of all detected transcripts with the list of predicted miR-501 targets in mouse and human, generated by TargetScan v6.2. Among the overlapping genes, those which were significantly upregulated (*P*<0.05) and were highly conserved among mammals were considered for further analysis (Fig. 6A; Fig. S1A,B). Importantly, we could confirm six of the selected genes as potential miR-501 targets as their expression decreased or increased after transfection of primary myoblasts with miR-501 mimics or antagomirs, respectively (Fig. 6B). In order to validate these genes as miR-501 targets, we cloned the part of their 3′UTRs that harbored the predicted miR-501-binding site in luciferase reporter vectors. Analysis of luciferase activity after co-transfection with a miR-501 mimic confirmed that all selected genes have at least one responsive miR-501 binding site (Fig. S1C). To investigate whether these genes underlie the effect of miR-501 on MYH3 expression in newly formed myofibers, we cloned the protein-coding sequence of the validated target genes into expression vectors. Overexpression in primary myoblasts was confirmed at the transcriptional level for all constructs (data not shown). For each construct, we then analyzed MYH3 protein levels 2 days after induction of differentiation. Overexpression of only one of the target genes, *Gan*, was able to reduce MYH3 levels in primary myoblasts (Fig. 6C), thereby mimicking the *in vivo* effect of inhibiting miR-501 in skeletal muscle. The downregulation of MYH3 after overexpression of *Gan* was rescued by the proteasome inhibitor MG-132 (Fig. 6D). These results are in line with reports of gigaxonin (also known as giant axonal neuropathy) being an E3 ligase substrate adaptor that targets proteins for proteasomal degradation and our findings that MYH3 is inhibited post-transcriptionally in the absence of miR-501. Luciferase assays using the 3′UTR of *Gan* harboring a mutated miR-501-binding site confirmed *Gan* as a direct miR-501 target (Fig. 6E). Inhibition of miR-501 using antagomirs increased *Gan* mRNA in regenerating muscle at day 4 after CTX, but not in MPs (Fig. 6F). Together, our results indicate that miR-501 is upregulated during muscle regeneration in myogenic progenitors and newly formed fibers where it regulates developmental myosins by its target gene *Gan*.

### miR-501 indicates muscle regeneration in the *mdx* mouse

**Fig. 7.**
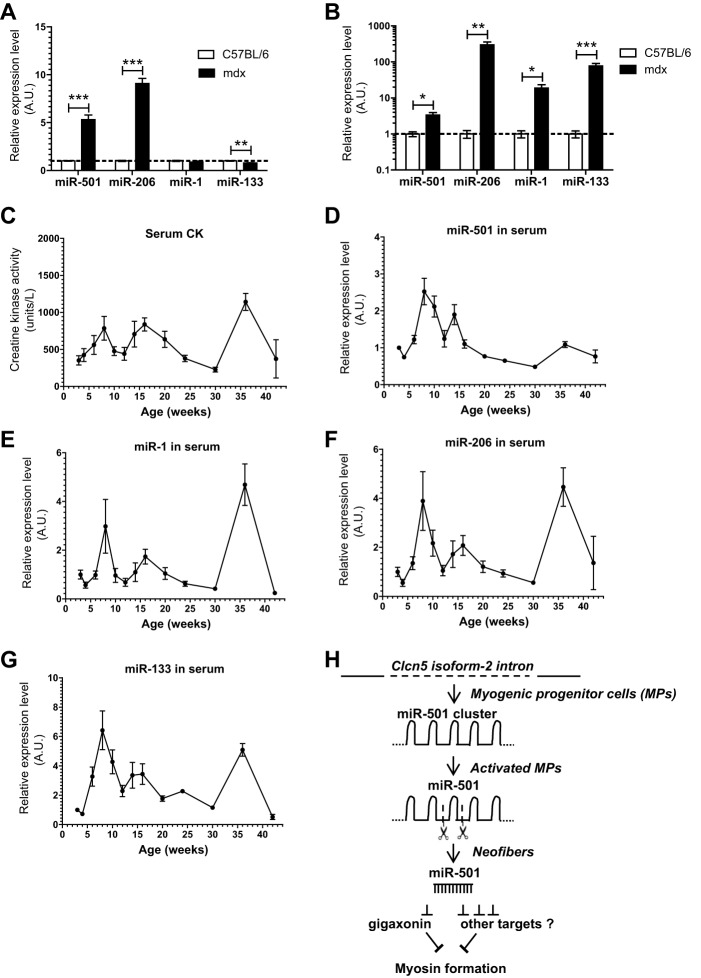
**miR-501 is increased in skeletal muscle from *mdx* mice and** **is** **a marker for disease severity in serum.** (A,B) Expression of miR-501 and myomiRs in TA muscle (A) or serum (B) from 12-week-old C57BL/6 and *mdx* mice as measured by qRT-PCR. Data are normalized to sno234 (*n*=3; A) or to the spiked cel-miR-39 (*n*=5-9; B). (C-G) Creatine kinase (CK) activity and expression of miR-501 and myomiRs in serum from *mdx* mice at different ages. miRNA expression levels were measured by qRT-PCR and normalized to the spiked cel-miR-39. *n*=3-10 per time point. (H) Hypothetical model for the expression and function of miR-501 during regeneration of adult muscle tissue. In MPs, the miR-501 cluster is transcribed together with *Clcn5* isoform-2. Muscle regeneration enhances the cleavage of the miR-501 cluster leading to increasing miR-501 concentrations in activated MPs and neofibers. In neofibers, miR-501 regulates myosin formation through gigaxonin and potentially other targets. Data are presented as mean±s.e.m. *P<0.05, **P<0.01, ***P<0.001, Student's *t*-test.

Our results so far identified a novel muscle-specific miRNA in a model of injury-induced muscle regeneration. We next addressed whether miR-501 is also upregulated in muscle from a genetic model of muscle regeneration, the *mdx* mouse. miR-501 expression was more than five times higher in tibialis anterior (TA) muscles from *mdx* mice than in those from age-matched wild-type C57BL/6 controls (Fig. 7A). As previously reported (Roberts et al., 2012), miR-206 levels were induced, whereas miR-1 and miR-133 were unchanged or downregulated in *mdx* mice compared with control (Fig. 7A). Next, we analyzed whether miR-501 was present in the serum of *mdx* mice and whether it correlated with the regeneration status of muscle, similar to previously reported myomiRs (Roberts et al., 2012). Comparison of miR-501 levels in serum from age-matched *mdx* and C57BL/6 mice showed significantly higher abundance of this miRNA and also miR-206, miR-1 and miR-133, in *mdx* mice (Fig. 7B). Time-course experiments revealed that the levels of all myomiRs including miR-501 correlated with serum creatine kinase activity (Fig. 7C-G). Serum miRNAs levels peak between 8 and 10 weeks of age, which is in line with the high proliferation/regeneration reported for the *mdx* mouse during this period (McGeachie et al., 1993; Turk et al., 2005; Zhou et al., 2006). Together, these results strengthen the view of miR-501 as a miRNA specific to muscle regeneration.

We therefore propose that miR-501 expression in muscle precursor cells is mediated by its host gene *Clcn5* isoform-2. During muscle regeneration, activation of MPs leads to post-transcriptional regulation and a surge in miR-501 expression in neofibers that supports myosin expression by targeting genes such as gigaxonin (Fig. 7H).

## DISCUSSION

Our study identifies a novel muscle-specific miRNA that regulates myosin during neofiber formation. miR-501 was only detectable in regenerating skeletal muscle and muscle progenitor cells, and loss-of-function studies using pharmacological inhibitors *in vivo* demonstrated a profound loss of MYH3 protein and to a lesser degree adult MHC after muscle injury was induced. Strikingly, an unbiased target identification approach in primary muscle cells validated gigaxonin as a target of miR-501, overexpression of which in differentiating muscle cells *in vitro* mimicked the effect of antagonizing miR-501 on myosin levels observed *in vivo*.

Gigaxonin is a ubiquitously expressed protein (Bomont et al., 2000) that acts as an E3 ligase adaptor and targets proteins for degradation by the proteaseome (Mahammad et al., 2013) in different cell types such as dermal fibroblasts (Sabatelli et al., 1992) and neurons (Ben Hamida et al., 1990). Mutations in the gene *Gan*, which encodes gigaxonin, can cause an autosomal recessive sensorimotor disease, called giant axonal neuropathy (Bomont et al., 2000), but a specific function for skeletal muscle has not been reported yet. Interestingly, in non-muscle tissues, gigaxonin targets intermediate filaments, such as vimentin (Sabatelli et al., 1992) or keratin (Treiber-Held et al., 1994) for proteasomal degradation, whereas in myofibers, we observed that gigaxonin did not affect the intermediate filament desmin, but instead the larger myosin.

The myosin composition of adult muscle changes depending on the physiological demands, especially during regeneration when MYH3 appears first and is later replaced by the adult isoforms. The effects of miR-501 inhibition were strongest on the newly formed myofibers and decreased as regeneration progressed and adult myofibrils were formed. These kinetics support the notion that miR-501 action involves inhibition of the proteasome. Several studies have shown that mature myofibrils are not degraded by the activated proteasome (Solomon and Goldberg, 1996) and additional enzymes are required to allow for the solubilization of ubiquitylated myofibrillar structures (Piccirillo and Goldberg, 2012). Therefore, during the early stages of regeneration, miR-501 might provide protection from the proteasome for the newly synthesized MYH3 protein as well as the MHC adult isoforms that are not incorporated yet into myofibrils. As myofibers mature and myofibrils form and stabilize, they are no longer targets for the proteasome and miR-501 is no longer required and is therefore downregulated. Interestingly, although the proteasome is responsible for the degradation of 80-90% of the muscle proteins *in vivo* (Goll et al., 2008), it is much less abundant in myotubes where proteolysis depends primarily on autophagy (Zhao et al., 2007). Indeed, inhibition or overexpression of miR-501 in primary muscle cells *in vitro* did not affect MYH3 levels (data not shown). The specific loss of a high molecular weight band that we observed in our SDS-PAGE analysis from muscle samples provides evidence against the involvement of the calpain system in the miR-501 pathway (Sugita et al., 1980).

The role of the transition of developmental myosins in muscle fiber regeneration is poorly understood. Our study indicates that it affects neofiber diameter although the effect size was subtle. It is possible that repeated CTX injections are required for more profound effects or that myosin transition has a stronger impact after a more physiological regeneration stimulus, e.g. after eccentric exercise training. The regulation of myosins during early myogenesis did not affect muscle histology 30 days after CTX injection when muscle regeneration is completed (Fig. S2). However, this does not exclude functional deficiencies. Indeed, the *miR-206* knockout mouse also showed a normal muscle architecture at day 30 after CTX injection, but displayed decreased muscle function during downhill running when miR-206 was genetically deleted in the chronic regeneration model, *mdx* (Liu et al., 2012). Future studies should address the consequences of the genetic deletion of miR-501 on exercise capacity. Targeting miR-501 could provide a unique opportunity to study the role of myosin heavy chain regulation in muscle regeneration.

Isoform-1 of the *Clcn5* gene encodes a Cl^−^/H^+^-exchanger highly expressed in kidneys where it plays a crucial role in the process of endocytosis in the proximal tubule (Piwon et al., 2000; Christensen et al., 2003). Mutations that alter *Clcn5* isoform-1 function can cause Dent's disease in humans, characterized by tubular proteinuria, hypercalciuria, nephrolithiasis, nephrocalcinosis and chronic renal failure (Devuyst and Thakker, 2010). The function of isoform-2 has not been reported, but based on its specific upregulation during muscle regeneration, this isoform deserves further functional characterization during this process. Assuming that isoform-2 is a functional chloride channel, it is possible that it might play a role in the cell cycle and proliferation of the muscle progenitors as hyperpolarization of stem cells has been reported to inhibit proliferation (Blackiston et al., 2009; Li and Deng, 2011).

Our studies confirm that miR-501 is a muscle-specific miRNA also based on its detection in skeletal muscle and serum from the *mdx* mouse. Circulating miR-501 levels correlate with the activity of skeletal muscle regeneration in *mdx* mice. MyomiRs have a great potential to be used as biomarkers for muscle diseases because their detection in serum is not confounded by contamination from blood or endothelial cells. Indeed, miR-1, miR-133 and miR-206 have been used to detect disease activity in muscle dystrophic animal models and patients (Cacchiarelli et al., 2011; Mizuno et al., 2011; Roberts et al., 2012, 2013). However, these inductions do not always correlate with the respective levels of the myomiRs in skeletal muscle itself as miR-1 and miR-133a were either decreased or unchanged in *mdx* muscle tissue (Mizuno et al., 2011; Roberts et al., 2012). In addition, miR-206 was shown to be already upregulated in serum from 2-week-old *mdx* mice, indicating that this miRNA can reach the circulation in the absence of muscle damage (Roberts et al., 2013). Moreover, miR-1 and miR-133a are also expressed in heart tissue, and miR-206 is preferentially expressed in type 1 muscle fibers (Liu et al., 2012). Hence, it is not clear whether the elevation of these previously known myomiRs in serum reflects muscle mass, muscle type, damage or regeneration. The specific expression of miR-501 in activated MPs of the skeletal muscle lineage presents a unique advantage for its use as a biomarker that deserves further testing in patient samples. The use of miR-501 as a biomarker in serum might also be extended to other clinical settings, for example to monitor the response of skeletal muscle to exercise. Because the number of activated MPs in skeletal muscle also increases during exercise interventions (Kadi et al., 2004; Dreyer et al., 2006; Petrella et al., 2008; Verney et al., 2008), non-invasive testing for the activation of MPs using miR-501 in serum could provide important information about the efficiency of different exercise protocols in humans.

miRNA expression has been previously studied in adult muscle stem cells using qRT-PCR-based miRNA microarrays (Cheung et al., 2012) or computational predictions (Farina et al., 2012). In the present study, we used RNA deep sequencing to identify novel muscle-specific miRNAs. The close genomic localization of miR-501 and miR-362 in mouse and human of <700 nucleotides and their similar expression pattern in MP and FAP indicates that they are transcribed as a miRNA cluster. MiRNAs that reside on polycistronic transcripts are processed with different efficiencies despite being co-transcribed (Chakraborty et al., 2012; Chaulk et al., 2014), which could explain the higher abundance of miR-501 compared with miR-362. We have recently described a similar phenomenon for the miR-29a/b cluster in mouse and human muscle cells (Galimov et al., 2016). We currently lack sufficient evidence to conclude that other miRNAs located in the Clcn5-2 intron also share the miR-362/-501 cluster. The strong induction of miR-501 upon activation of MPs is not observed for its host gene *Clcn5* isoform-2. This disconnection between the abundance of the host gene and the mature miRNA levels is not clear, but is consistent with the complex regulation of polycistronic miRNA transcripts (Chakraborty et al., 2012; Chaulk et al., 2014). Although we cannot exclude the possibility that the miR-362/-501 cluster has an independent promoter, the striking co-expression of Clcn5-2 and miR-501 during muscle regeneration still favors a shared promoter region for the two genes.

The effect size for the upregulation of gigaxonin mRNA after miR-501 inhibition during muscle regeneration *in vivo* was small. However, gigaxonin is ubiquitously expressed and might reach much higher expression levels in neurons and nerve endings compared with muscle, which might dilute the observed effects after inhibition of miR-501. Because a single miRNA usually targets many different genes, it is also likely that there are other targets of miR-501 than gigaxonin yet to be discovered. Further studies that employ sequencing studies *in vivo* should reveal additional targets of miR-501 also involved in muscle regeneration and the formation of myosin.

In conclusion, we found that the novel myomiR 501 is a critical checkpoint during muscle regeneration to prevent the expression of genes such as gigaxonin that are disadvantageous for the newly formed myofiber. Unraveling this pathway might lead to new pharmacological strategies to enhance muscle regeneration and prevent muscle wasting in disease states such as cachexia or aging.

## MATERIALS AND METHODS

### Mice

C57BL6/6J or C57BL/10ScSn-Dmd*^mdx^* mice were purchased from Harlan or Jackson Laboratories, respectively. Animals were housed in a pathogen-free animal facility in University Hospital Zurich on an inverted 12-h light cycle (dark phase began at 06.00 h), fed *ad libitum* with chow diet. All animal studies were approved by the ethics committee of the Canton of Zurich Veterinary Office. Guidelines set by the Swiss Federal Veterinary Office were followed in all procedures.

### Cardiotoxin and antagomir injection

Cardiotoxin from *Naja mossambica mossambica* (Sigma-Aldrich) was dissolved in PBS to a final concentration of 10 µM and injected into C57BL6/6J mouse TA muscles at 50 µl per muscle. Antagomir (7.5 µg) was injected at the indicated time points (50 µl per muscle). Antagomirs were ordered in full-length complementarity to miR-501 with the previously described modifications (Krützfeldt et al., 2005) from Sigma-Aldrich. For tissue harvesting, TA muscles were dissected, snap-frozen in liquid nitrogen and stored at −80°C.

### Measurement of creatine kinase activity and serum collection

Mice were euthanized by CO_2_ inhalation, blood was collected using 25G syringes from heart ventricles and transferred to 1.5 ml eppendorf tubes to coagulate. Tubes were centrifuged at 2000 ***g*** for 8 min and the supernatant was again centrifuged at 12,000 ***g*** for 15 min to precipitate the residual cell debris. Serum creatine kinase activity was measured using a Creatine Kinase Activity Assay Kit (Sigma-Aldrich) according to the manufacturer's instructions.

### Isolation of primary myogenic and fibro/adipogenic progenitors

Mouse TA muscles were minced in cold PBS on ice and digested with collagenase type II (Life Technologies). Resulting cells were suspended in 0.5% bovine serum albumin in PBS and incubated with the respective antibodies on a rotating chamber at 4°C. 7-AAD (7-aminoactinomycin D; A9400, Sigma-Aldrich) was used to exclude dead cells. The following antibodies were used: Alexa Fluor 488 anti-mouse CD45 (clone 30-F11, Biolegend), Alexa Fluor 488 anti-mouse CD31 (clone 390, Biolegend), APC anti-mouse SCA1 (clone D7, Biolegend) and PE anti-mouse alpha7-integrin (FAB3518P, R&D Systems). Sorting was performed on a FACSAria III (BD Biosciences).

### Analysis of proliferation of muscle-resident progenitors

Twelve hours before harvesting the muscle tissue, mice were injected with 10 µg/g bodyweight EdU intraperitoneally. After collagenase digestion, cells were subjected to staining for detection of the incorporated EdU using the Click-iT EdU Alexa Fluor 647 Flow Cytometry Assay Kit (Life Technologies) according to the manufacturer's instructions.

### Culture and transfection of primary myoblasts and HEK 293 cells

Cells were cultured on collagen-coated plates in growth media, consisting of 40% (v/v) low-glucose DMEM, 40% (v/v) HAM's F-10 medium, 20% fetal bovine serum, 5 ng/ml basic fibroblast growth factor, and 100 U/ml Penicillin/Streptomycin (all from Life Technologies). To induce differentiation, myoblasts were transferred to differentiation media (low-glucose DMEM supplemented with 2% horse serum and 100 U/ml Penicillin/Streptomycin) after reaching 80% confluency. Lipofectamine RNAiMax was used for transfecting miRNA mimics (MISSION miRNA, Sigma-Aldrich) or antagomirs to the cells. Final concentration was 38 nM for mimics and 12 nM for antagomirs, respectively. Lipofectamine LTX was used for DNA transfection, and co-transfection of DNA with miRNA mimics or antagomirs was performed using Lipofectamine 2000. All reagents were purchased from Invitrogen, and transfection was performed according to the manufacturer's instructions. MG-132 (474790) was obtained from Merck Millipore. HEK 293 cells were cultured in high-glucose DMEM supplemented with 10% fetal bovine serum and 100 U/ml Penicillin/Streptomycin (all from Life Technologies). Lipofectamine 2000 (Invitrogen) was used for co-transfection of DNA with miRNA mimics or antagomirs, according to the manufacturer's instructions.

### RNA isolation and qRT-PCR

Total RNA was isolated using Trizol reagent (Invitrogen) according to the manufacturer's instructions. RNA Clean-Up and Concentration Kit (Norgen Biotek) was used for RNA extraction from sorted myogenic progenitors. Isolation of RNA from serum was performed using miRNeasy Mini Kit (Qiagen) from 50 µl serum added to 700 µl Qiazol (Qiagen) and spiked with cel-miR-39 as an internal control, according to the manufacturer's instructions. After using the RNase-free DNase I Kit (Ambion), RNA was precipitated in 70% ethanol containing 0.1 M ammonium acetate at −80°C overnight. First-strand cDNA was synthesized using SuperScript III Reverse Transcriptase Kit (Thermo Fisher Scientific) with random hexamers according to the manufacturer's instructions. FastStart Universal SYBR Green Master (Roche) was used to run the real-time PCR reactions on a 7500 Fast Real-Time PCR system (Applied Biosystems). Primer sequences are listed in Table S1. Three primer sets were designed for measuring *Clcn5* gene expression: a general primer set (F_C_ and R_C_), which amplifies both *Clcn5* splice variants, a primer set specific for isoform 1 (F_1_ and R_1,2_), and a primer set specific for isoform 2 (F_2_ and R_1,2_) as indicated in Fig. 3A. First, two PCR reactions were performed on a mouse cDNA sample with either F_1_ and R_C_, or F_2_ and R_C_ primers. PCR products were purified and named Iso1-std and Iso2-std, respectively. As the binding site for R_C_ primer is downstream of that of R_1,2_, each of the resulting PCR products contained the part that could be amplified by the general primers (F_C_ and R_C_) preceded by their corresponding isoform-specific sequence. Serial dilutions of Iso1-std and Iso2-std were used as standard samples to quantify isoform-1 or isoform-2 transcripts, respectively, using the isoform-specific primers. In a separate reaction, using F_C_ and R_C_ primers, the Iso1-std serial dilutions were measured against serial dilutions of Iso2-std as standards. This enabled adjustment of the absolute copy numbers ascribed to the Iso1-std serial dilutions, and consequently scaling of the values obtained for isoform 1 to those for isoform 2. For quantification of miRNA levels, first-strand cDNA synthesis and qPCR reactions were performed using TaqMan MicroRNA Reverse Transcription Kit (Applied Biosystems) and TaqMan Universal PCR Master Mix (Thermo Fisher Scientific), respectively.

### RNA sequencing

RNA sequencing was carried out as a service by LC Sciences (Houston, TX, USA). Expression of miRNAs were measured by the relative frequency of reads assigned to each miRNA and stated as RPM (reads per million). For mRNA sequencing, miRNeasy Mini Kit (Qiagen) was used, accompanied by on-column DNase-treatment according to the manufacturer's instructions. Poly (A) enrichment, library preparation and single-end sequencing of 100-nucleotide sequences on Illumina HiSeq-2000 was performed as a service by the Functional Genomics Center Zurich (FGCZ). Mapping of raw sequence reads to the genome was performed using bowtie program with mouse genome build mm10 as reference. Transcript quantification and differential expression analysis were performed using RSEM and edgeR software, respectively, on Bioconductor package in R. FastQC program in Java was used for quality control of the high-throughput data.

### Northern blotting

Polyacrylamide gels were used to separate 15 µg of total RNA. RNA bands were visualized by incubating the gels after electrophoresis in 0.5 µg/ml ethidium bromide and RNA was transferred onto Hybond+ membranes (Amersham). The membrane was dried and cross-linked under UV radiation (1200 J/m^2^) and hybridized with a radioactively labeled probe (γ-^32^P-ATP, 10 mCi/ml). The washed membrane was exposed to a phosphorimager to detect the radioactivity signal. Signal density of the bands was measured using ImageJ (NIH).

### Western blotting

Cells were lysed in RIPA buffer (25 mM Tris HCl pH 7.6, 150 mM NaCl, 1% NP-40, 1% sodium deoxycholate, 0.1% SDS) supplemented with complete EDTA-free protease inhibitor cocktail and PhosSTOP phosphatase inhibitor cocktail (Roche). SDS-PAGE was performed on NuPAGE Novex 4-12% Bis-Tris protein gels, and separated with SDS running buffer at 120 V. The gel was then used for Coomassie Blue staining or western blotting to PDVF membrane using Mini Trans-Blot Cell chambers (Bio-Rad). Membranes were incubated at 4°C with the primary antibodies for at least 16 h [anti-total myosin heavy chain from DSHB, MF-20 (1:100), anti-myosin heavy chain-3 from Santa Cruz (sc-53091; 1:500), anti-desmin from Sigma (D1033; 1:200), anti-GAPDH from ProteinTech (10494-1-AP; 1:1000), anti-gigaxonin from Proteintech (14305-1-AP; 1:500)]. Incubation with appropriate HRP-conjugated secondary antibodies and the Lumi-Light western blotting substrate (Roche) was used to detect the signal in a LAS-3000 imager (Fujifilm). Densitometry analysis of the images was carried out using Aida image analyzer software v.2.3.

### Luciferase activity assay

The 3′UTR fragments of the selected genes were amplified by PCR from human muscle cDNA using high fidelity PCR enzyme mix (Thermo Scientific). PCR products were cloned in pCR2.1 vector using TOPO TA cloning kit (Life Technologies) and subcloned into pmirGLO Dual-Luciferase miRNA Target Expression Vector (Promega), downstream of the firefly luciferase open reading frame (ORF). Mutagenesis of the miR-501 seed motif in the 3′ UTR of *Gan* was performed using the QuikChange II Site-Directed Mutagenesis Kit (Agilent Technologies). To make the miR-501 sensor construct, two pairs of oligonucleotides each pair harboring a full complementary biding site for miR-501, were annealed and cloned into the *Xba*I site after firefly luciferase ORF in pmirGLO vector. DNA constructs were transfected to HEK 293 cells or primary myoblasts along with miRNA mimics or antagomirs using Lipofectamine 2000 (Invitrogen) according to the manufacturer's instructions. Final concentrations of DNA, miRNA mimic and antagomirs were 200 ng/ml, 38 nM and 12 nM, respectively. Activity of Firefly and *Renilla* luciferase enzymes was measured using Dual-Luciferase Reporter Assay System (Promega) with a Tecan plate reader.

### Generation of overexpression DNA constructs

A double-stranded DNA molecule containing the Kozak consensus sequence including start codon (ATG) followed by a sequence encoding the FLAG-tag (Asp-Tyr-Lys-Asp-Asp-Asp-Asp-Lys) was made by annealing two oligonucleotides and ligated into pcDNA3.1 (+) mammalian expression vector (Invitrogen). The resulting construct (pcDNA-Kozak-Flag) was used to make overexpression constructs encoding for FLAG-tagged proteins. ORFs of the respective genes were amplified by PCR from mouse multi-tissue cDNA using high fidelity PCR enzyme mix (Thermo Scientific) and specific primers. Primer sequences are listed in Table S1. A *Hin*dIII restriction site was included at the end of both forward and reverse primers. PCR products were digested with *Hin*dIII enzyme and cloned in the *Hin*dIII site of the pcDNA-Kozak-Flag vector. All constructs were confirmed by restriction mapping and DNA sequencing.

### Skeletal muscle immunofluorescence

Skeletal muscle was dissected and flash frozen in isopentane/liquid nitrogen. Frozen sections of 10 µm were prepared from three different areas of the tibialis anterior muscle, 500 µm apart. Sections were then processed for immunofluorescence using the Vector M.O.M. Immunodetection Kit and protocol (Vector Laboratories). Slides were mounted with Fluoroshield with DAPI (Sigma). Antibodies against eMHC (BF-G6, 1:20) were obtained from the Developmental Studies Hybridoma Bank, University of Iowa and antibodies against laminin (L9393, 1:500) were obtained from Sigma. Images for each section were obtained using Leica confocal laser scanning microscope SP5 Mid UV-VIS and evaluated for fiber and cell counts using Ilastic and ImageJ software.

### Statistical analysis

Unless specified otherwise, two-sided unpaired Student's *t*-test was used to compare sample groups, and the null hypothesis (no difference between the groups) was rejected if *P*<0.05. When applicable, one-sample *t*-test with a hypothetical mean value of 1 was used to analyze the significance based on fold-change values. Differential expression analysis of RNA-seq data was performed by edgeR software. Data in bar graphs are shown as mean±s.e.m.
